# D-ribose metabolic disorder and diabetes mellitus

**DOI:** 10.1007/s11033-023-09076-y

**Published:** 2024-01-28

**Authors:** Yu Tai, Zehong Zhang, Zhi Liu, Xiaojing Li, Zhongbin Yang, Zeying Wang, Liang An, Qiang Ma, Yan Su

**Affiliations:** 1 Institute of Biochemistry and Molecular Biology, Baotou Medical College, Baotou, Inner Mongolia China; 2 Department of Clinical Laboratory, the Fourth Hospital of Baotou, Baotou, Inner Mongolia China

**Keywords:** D-ribose, Diabetes mellitus, Nonenzymatic glycation, Advanced glycation end products, Energy metabolism

## Abstract

D-ribose, an ubiquitous pentose compound found in all living cells, serves as a vital constituent of numerous essential biomolecules, including RNA, nucleotides, and riboflavin. It plays a crucial role in various fundamental life processes. Within the cellular milieu, exogenously supplied D-ribose can undergo phosphorylation to yield ribose-5-phosphate (R-5-P). This R-5-P compound serves a dual purpose: it not only contributes to adenosine triphosphate (ATP) production through the nonoxidative phase of the pentose phosphate pathway (PPP) but also participates in nucleotide synthesis. Consequently, D-ribose is employed both as a therapeutic agent for enhancing cardiac function in heart failure patients and as a remedy for post-exercise fatigue. Nevertheless, recent clinical studies have suggested a potential link between D-ribose metabolic disturbances and type 2 diabetes mellitus (T2DM) along with its associated complications. Additionally, certain in vitro experiments have indicated that exogenous D-ribose exposure could trigger apoptosis in specific cell lines. This article comprehensively reviews the current advancements in D-ribose’s digestion, absorption, transmembrane transport, intracellular metabolic pathways, impact on cellular behaviour, and elevated levels in diabetes mellitus. It also identifies areas requiring further investigation.

## Introduction

Ribose (C_5_H_10_O_5_), with a molecular weight of 150.13 Dalton, is a pentose sugar comprising two enantiomers: L-ribose and D-ribose. D-ribose, as the more stable form of the two enantiomers, serves as the predominant functional isoform found in all living cells [1, 2]. D-ribose assumes a pivotal role as a constituent in various critical biomolecules, including RNA and nucleotides. It plays a fundamental role in processes governing cell growth, division, development, and reproduction, contributing significantly to essential life activities. However, recent studies have raised the possibility of an association between elevated D-ribose levels and certain medical conditions, such as diabetes and cognitive dysfunction [1, 3]. These seemingly contrasting findings underscore the need for a deeper comprehension of D-ribose's biological functions, metabolism, and cytotoxic potentials. Such insights may offer fresh perspectives on the pathogenesis of these relevant diseases.

## Absorption, distribution, and transmembrane transport of D-ribose in the body

### Absorption and distribution of D-ribose

Exogenous D-ribose primarily derives from dietary sources rich in RNA and riboflavin, while endogenous D-ribose is chiefly biosynthesized from glucose through the pentose phosphate pathway (PPP). The European Food Safety Authority (EFSA) has affirmed that D-ribose is safe for the general population, even at daily intake levels of up to 36 mg/(kg·bw). The minimum threshold for adverse effects in adult individuals was recorded at a daily intake of 10 grams [4, 5].

A significant proportion (ranging from 87.8 to 99.8%) of D-ribose in food and pharmaceuticals is absorbed via the intestinal tract and enters the bloodstream [6]. In healthy adults, the plasma concentration of D-ribose hovers between 0.02 to 0.1 mM, markedly lower than that of glucose, which ranges from 3.9 to 6.1 mM [7]. However, comprehensive studies examining the cellular and tissue distribution of D-ribose were relatively scarce.

One early investigation dating back to 1958 utilized the isotope ^14^C to trace D-ribose and assessed its blood clearance rates in seven healthy patients and three diabetic patients [8]. The findings revealed that approximately 21% of ^14^C-D-ribose was excreted in urine within 15 min after D-ribose injection, and almost all of injected D-ribose was eliminated from urine within 90 min. The clearance rates of ^14^C-D-ribose were found to be identical between healthy and diabetic patients. Furthermore, insulin administration was observed to expedite the clearance of D-ribose from the bloodstream, leading to a swift reduction in blood glucose levels following D-ribose injection. It's worth noting that this study was conducted on humans in vivo and solely examined the blood clearance of D-ribose, without insights into its distribution in other tissues or organs.

Subsequently, Gaitond and Arnfred studied the distribution of 14C-D-ribose in the blood, liver, heart, and brain of rats [9]. Their observations indicated that ^14^C-D-ribose was rapidly absorbed by the brain and liver within 5–60 min of administration, while insulin notably enhanced the clearance of D-ribose from the bloodstream, but had no discernible effect on its entry into muscle tissue.

In 2014, Clark’s investigation highlighted considerable tissue specificity of D-ribose distribution in mice, with a predominant accumulation in the liver [10]. More recently, a pharmacokinetic experiment involving intravenous injection and oral administration of D-ribose was conducted on healthy rabbits. The results illustrated rapid absorption by the digestive tract, followed by a swift decline in plasma D-ribose levels. Intriguingly, only 18% to 37.5% of the administered D-ribose was excreted in urine, implying that a portion of D-ribose may indeed permeate into various tissues [11]. However, the available literature on D-ribose absorption and excretion in rats remains limited. Existing data does indicate that D-ribose is rapidly and nearly completely absorbed in humans when administered at a rate of 200 mg/(kg·bw) per hour over 5 h, with the excretion percentage increasing as the dose escalates [4].

### Tansmembrane transport of exogenous D-ribose

The utilization of exogenous D-ribose hinges upon its transmembrane transport through the plasma membrane; however studies scrutinizing this process have been relatively scarce. An intriguing clue emerged when a variant of the Novikoff hepatoma cell line exhibited the capability to employ D-ribose as its exclusive source of carbon and energy, hinting at the potential for mammalian cells to uptake D-ribose [12]. In 2003, Lager’s investigation suggested that the absorption of D-ribose in COS-7 cells might be facilitated by the glucose transporter (GLUT) family [13].

Subsequent research by Naula revealed that LmGT2, a GLUT within the protozoan parasite *Leishmania*, displayed homology with the mammalian GLUT family and acted as an effective carrier of D-ribose [14]. The human body houses no Fewer than 14 distinct GLUT variants in various tissues. Clark and colleagues illuminated that GLUT2 had the capacity to usher a portion of D-ribose into hepatic cells, shedding light on the transport mechanism within the liver. However, the specific transporters responsible for D-ribose uptake in other tissues remain enigmatic, warranting further exploration.

It is worth noting that 3-O-methyl-D-glucose, possessing a structural likeness to D-glucose, is transportable into cells through GLUT but eludes glycolytic metabolism. Consequently, it serves as a valuable tool for scrutinizing glucose transport and metabolic processes. Some studies have examined how D-ribose influences the transport of 3-O-methyl-D-glucose into diverse tissue cells. The findings indicated that D-ribose had no discernible inhibitory impact on rat hepatocytes [15], but competitively impeded the uptake of 3-O-methylglucose in primary cultured bovine brain microvascular endothelial cells [16]. These outcomes underscore the potential divergence in transport mechanisms for D-ribose and D-glucose across various cells.

## Intracellular metabolic pathways of D-ribose

**Fig. 1:**
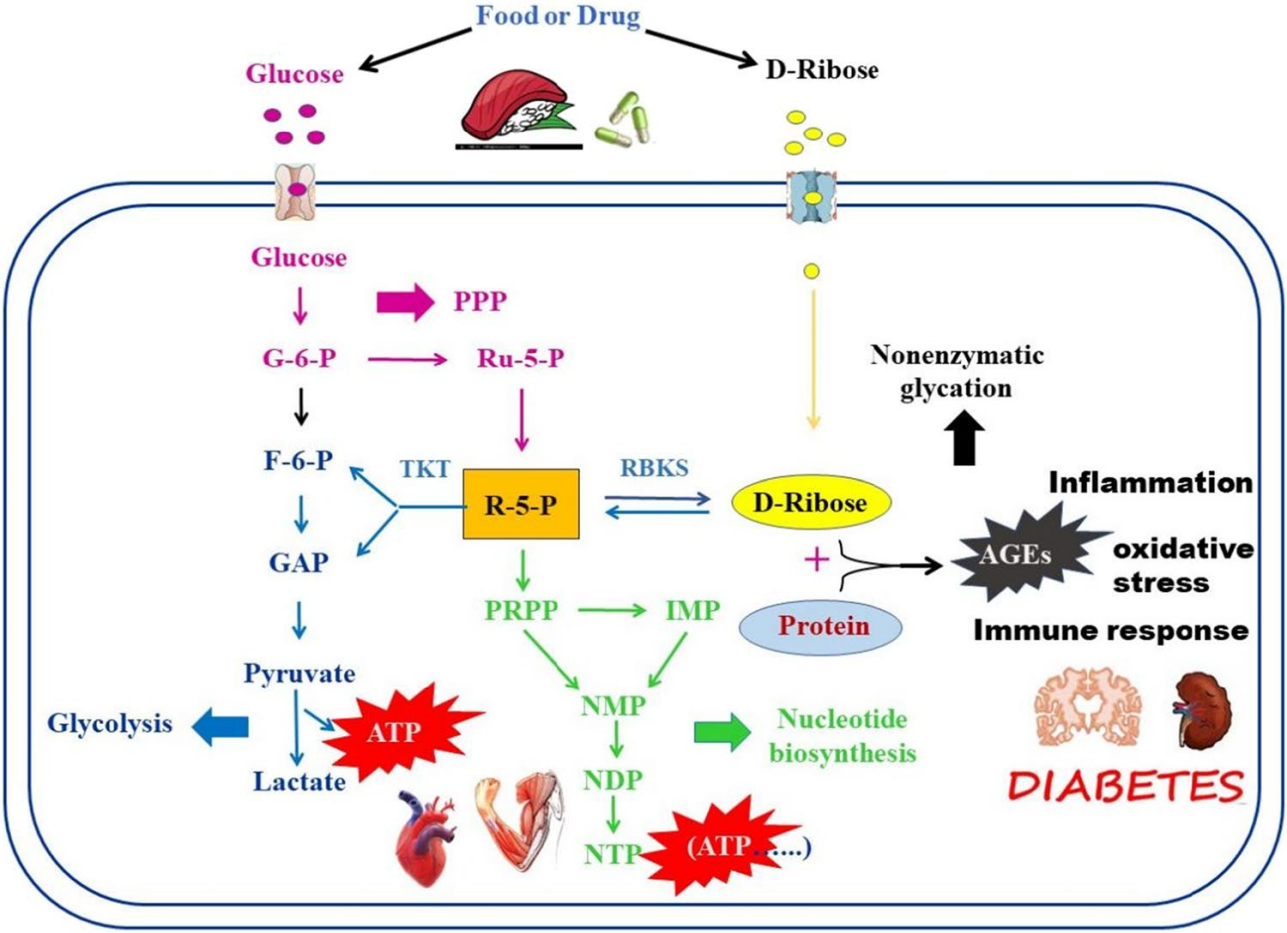
The intracellular metabolism of endogenous and exogenous D-ribose. Exogenous D-ribose primarily derives from food or drug, while endogenous D-ribose is mainly biosynthesized from glucose through the pentose phosphate pathway (PPP). The reversible transformation between D-ribose and R-5-P can be catalyzed by RBKS. When the intracellular D-ribose level increases, some of them may enter into the nonoxidative stage of PPP, ultimately generating F-6-P and G-3-P to produce ATP through anaerobic glycolysis or aerobic glucose oxidation; the other may be catalyzed to PRPP to participate in the nucleotide (including ATP) synthesis. Both of the above pathways can supply ATP for the cells to facilitate their growth. However, when excessive D-ribose is deposited in the cell, it can also initiate rapid nonenzymatic glycation reactions AGEs, which can cause damage to the cells

D-ribose, in general, is synthesized from glucose via the PPP within the cell. This process begins with D-glucose as a precursor for D-ribose synthesis, which undergoes phosphorylation to form glucose-6-phosphate (G-6-P). Subsequently, G-6-P is oxidized to 5-phosphate ribulose (Ru-5-P) along with NADPH through the oxidative phase of the PPP, as illustrated in Fig. 1. Following this, the resulting Ru-5-P is isomerised into ribose-5-phosphate (R-5-P), offering two metabolic routes.

One of these metabolic pathways segues into the nonoxidative stage, marked by a series of group transfer reactions within the PPP, ultimately generating fructose-6-phosphate (F-6-P) and glyceraldehyde-3-phosphate (G-3-P). These compounds serve as intermediate metabolites in the glycolytic pathway and can be further oxidized through either anaerobic glycolysis or aerobic glucose oxidation to yield ATP [17]. The second pathway is catalysed by phosphoribosyl pyrophosphate (PRPP) synthase, resulting in the formation of PRPP. This molecule plays a pivotal role in subsequent nucleotide synthesis, being involved in both de novo synthesis and salvage synthesis pathways. Externally sourced D-ribose, derived from dietary intake and pharmaceuticals, can permeate cellular membranes and then be phosphorylated to R-5-P by ribokinase, an ATP-dependent sugar kinase responsible for orchestrating D-ribose's entry into cellular metabolism [18]. From this point onward, R-5-P follows the same metabolic trajectories as delineated above.

## Effect of exogenous D-ribose on cells

### Cytoprotective effect of exogenous D-ribose on skeletal muscle and cardiomyocytes

Since the 1970s, numerous studies have unveiled D-ribose not only as a vital genetic material constituent but also as an energy source for myocardium and skeletal muscles. This feature is likely associated with the distinct energy metabolism of these muscle types. Glucose, as the primary substrate for ATP synthesis, undergoes a complex and time-consuming synthesis process. Furthermore, the rate of adenosine monophosphate (AMP) and ATP production from glucose varies across different organs, with the kidney having the highest production rate, followed by the liver, and heart, while skeletal muscles exhibit the lowest rate. Consequently, the myocardium and skeletal muscles are most susceptible to damage when ATP synthesis is insufficient. Supplementation of D-ribose can rapidly provide substrates for AMP and ATP synthesis. This not only enhances the body’s physical performance [19, 20] but also improves myocardial diastolic function in patients with ischemic cardiovascular disease and congestive heart failure, thus enhancing the heart’s resilience to ischemia [21–26]. Additionally, owing to its strong reductive properties, D-ribose can scavenge oxygen free radicals, bolster the body’s antioxidative capacity, curb membrane lipid peroxidation, and thus preserve membrane structure and function. Furthermore, Addis [27] suggested that combined D-ribose and antioxidant supplementation may exert cytoprotective effects during and after oxidative stress by influencing the release of superoxide anion free radicals. However, Faller’s study [28] reached a different conclusion, noting that increased D-ribose content in mouse myocardium did not maintain total adenine nucleotide (TAN) pools, nor did it enhance left ventricular function following myocardial infarction. A recent review article by Antonella et al. [29] proposed that due to structural unreliability, D-ribose serves as an ineffective precursor of R-5-P and thus may not significantly contribute to nucleotide pool replenishment. Further research is necessary to determine the optimal dose of D-ribose supplementation for beneficial effects and to identify any potentially harmful thresholds.

### Cytotoxicity effect of exogenous D-ribose

#### Strong nonenzymatic glycosylation ability of D-ribose

Nonenzymatic glycosylation (NEG), commonly known as glycation, denotes the process in which the aldehyde groups of reducing sugars, such as glucose, engage with the free amino groups present in macromolecular substances, including proteins, amino acids, lipids, and nucleic acids. This interaction forms reversible Schiff's bases and Amadori products. When subjected to nonenzymatic conditions and processes like oxidation, rearrangement, and cross-linking, these products can evolve into irreversible advanced glycation end products (AGEs) [30, 31]. D-ribose, owing to its chemical reactivity, exhibits a robust capacity for nonenzymatic glycation. Mou et al. [32] conducted an experiment in which they exposed BSA to a 20 mM D-ribose solution for 14 days, subsequently employing liquid chromatography-mass spectrometry to identify the ribosylation sites. They discovered that D-ribose glycated 59.9% of protein residues. These glycated protein residues encompass arginine pyrimidine (1.4%), carboxymethyl lysine (CML) (34.3%), carboxyethyl lysine (CEL) (7.1%), CML + CEL (15.7%), and CEL + pyrrolidine (1.4%). Seventeen of these lysine residues were selectively modified by D-ribose. Computational predictions of glycation sites indicated that D-ribose interacted with fibrinogen through three amino acid residues, namely arginine 104, aspartate 105, and alanine 101 [33]. In the case of human myoglobin (HMb), D-ribose rapidly induces protein glycation at lysine residues K34, K87, K56, and K147 located on the protein's surface [34]. Regarding albumin, K36, K75, K88, K161, K186, K229, K264, K286, K300, K341, K383, K396, K402, K437, K438, K597, and K598 are all subjected to glycosylation with ribose, while only K88, K161, K186, K300, K341, K438, and K499 experience glycosylation with glucose [35]. Among the ribosylated lysine residues in albumin, K36 and K438 have links to prediabetes [35]. Relative to glucose, mannose, galactose, xylose, fructose, and arabinose, D-ribose exhibits the highest nonenzymatic glycation ability on albumin [36]. Research by Chen [37] confirmed D-ribose’s involvement in the nonenzymatic glycation of serum proteins. Notably, D-ribose glycation of serum proteins was both more pronounced and faster compared to D-glucose, further underscoring D-ribose’s heightened nonenzymatic glycation capability. Multiple studies have pointed out that D-ribose can interact with Hb [6, 38–40], myoglobin [34], bovine serum albumin (BSA) [35, 37, 41], fibrinogen [42], β2-microglobulin [43], α-synuclein [44], and human immunoglobulin-G [45], thereby initiating rapid nonenzymatic glycation reactions and protein aggregation. Glycation with D-ribose leads to structural alterations in native Hb [46]. Glycyrrhizic acid [47], iridin [48], and phytochemical thymoquinone [39] are reported to counteract D-ribose-mediated protein glycation.

#### Ribosylation products and their autoantibodies

Glycated hemoglobin A1c (HbA1c) and glycated serum protein (GSP) stand as the primary diagnostic biomarkers employed for both diagnosis and treatment decisions for diabetic complications [49, 50]. Recent research has revealed that D-ribose induces structural alterations in Hb, triggers immune responses, and gives rise to autoantibodies directed at ribosylated Hb. These autoantibodies exhibit significant epitopes and are notably elevated in type 2 diabetes mellitus (T2DM) patients [7]. The inhibition of autoantibody production has the potential to decelerate the progression of diabetes and its related complications. Akhter et al. [51, 52] illustrated the presence of ribosylated low-density lipoprotein (LDL) autoantibodies in individuals with diabetes and atherosclerosis. Similar to glycated Hb, ribosylated LDL undergoes structural changes, displays heightened antigenic reactivity, generates neoantigenic epitopes, and stimulates the immune system to produce autoantibodies. The presence of anti-D-ribose-LDL autoantibodies in the serum of individuals with type 1 diabetes mellitus (T1DM) and T2DM may stem from prolonged autoimmune responses to LDL-AGEs. Furthermore, it has been observed that D-ribose glycosylates DNA, significantly altering its structure and inducing immune responses marked by high titer antibodies [53]. Consequently, the glycation of D-ribose within the body can incite an immune response, exacerbating the progression of diabetes. As a result, these autoantibodies possess the potential to serve as biomarkers for diabetes and its associated complications.

#### Cytotoxicity effects of exogenous D-ribose

A subsequent series of in vitro experiments has substantiated that elevated concentrations of D-ribose exert detrimental effects on peripheral blood mononuclear cells, human neuroblastoma SY5Y cells, glomerular interstitial cells SV40 MES 13, human glioma cells U251, human astroblastoma cells U87MG, renal mesangial cells, and Chinese hamster ovary (CHO) cells [38, 54, 55]. The toxicity mechanism of D-ribose is primarily associated with AGEs, which comprise a diverse and highly reactive group of compounds. The interaction of AGEs with their primary cellular receptor, RAGE, activates multiple signalling pathways, including MAPK/ERK, TGF-β, JNK, and NF-κB, culminating in escalated oxidative stress and inflammation [56]. These processes are linked to various maladies such as diabetes, kidney disease, Alzheimer’s disease, and cataracts [57–60].

Furthermore, the alterations in the structure and function of D-ribose glycated products are notably more pronounced than those observed in D-glucose nonenzymatic glycosylation products. Consequently, D-ribose exhibits more potent and rapid cytotoxicity towards in vitro cultured cells when compared to D-glucose [61].

## Elevated D-ribose levels in diabetes mellitus

### Elevated serum and urine D-ribose levels in diabetic patients

Diabetes mellitus (DM) is a progressive metabolic disorder characterized by disturbances in glucose metabolism and sustained chronic hyperglycemia [62]. Recent studies have indicated that urinary D-ribose levels in individuals with T2DM are significantly higher than those in healthy individuals, in addition to D-glucose [63]. Consequently, it is plausible that T2DM may not only manifest abnormal glucose metabolism but also abnormal D-ribose metabolism. Clinical investigations by Yu have demonstrated that urine and serum D-ribose levels are substantially elevated in T1DM patients, with these results subsequently confirmed in animal experiments [64]. Well-established clinical indicators for diabetes diagnosis including HbA1c and GSP. HbA1c reflects blood glucose levels over the past 2–3 months [65], while GSP specifically reflects blood glucose levels from the past 1–3 weeks [6]. Chen's study found a positive correlation between the urinary D-ribose levels of T2DM patients and their plasma HbA1c levels. Furthermore, in streptozotocin-induced diabetic rats and T2DM patients, plasma D-ribose levels positively correlated with GSP levels. The strength of the positive correlation between D-ribose and GSP exceeded that between D-glucose and GSP, suggesting that D-ribose plays a significant role in the generation of HbA1c and GSP [49]. Yoo et al. demonstrated that usage of the ribosylated fetal bovine serum reduced the proliferation of islet beta cells, increased cell apoptosis and necrosis, and subsequently impacted insulin secretion [66].

### D-ribose accelerates the progression of diabetes complications

Diabetes complications represent the primary cause of mortality and disability among diabetic patients [67]. Persistent hyperglycaemia in these individuals contributes to increased nonenzymatic glycosylation of proteins, thereby exacerbating various diabetic complications. Iannuzzi et al. [68] revealed that ribosylation did not alter the conformation of insulin, yet ribosylated insulin significantly impairs cell viability. This process activates the apoptotic pathway and triggers Caspase 9, 3 and 7, leading to the activation of the transcription factor NF-κB and the subsequent generation of intracellular reactive oxygen species (ROS). The detrimental impact of ROS on surrounding tissues and cells is acknowledged to be a primary cause of chronic diabetes complications. Presently, much of the related research focuses on diabetic nephropathy (DN) and diabetic encephalopathy.

#### Diabetic nephropathy

DN represents a severe microvascular complication of diabetes, manifesting in approximately 40% of patients with T2DM within 10 years of diagnosis [69]. Extended exposure to a hyperglycaemic environment results in the increased generation and accumulation of AGEs in vital organs, including the kidneys. AGEs have the potential to activate the mitogen-activated protein kinase/extracellular regulated protein kinase (MAPK/ERK) pathway and AGEs/ receptor of AGEs signalling, consequently inciting oxidative stress, endoplasmic reticulum stress, inflammation, fibrosis, and accelerating pathological renal alterations. These findings underscore the close relationship between AGEs and diabetic nephropathy [70].

AGEs generated by D-ribose contribute to glomerular cell dysfunction. Recent studies have demonstrated that prolonged D-ribose administration triggers the formation and activation of the NLRP3 inflammasome in podocytes through the AGEs/ribosylated AGEs (RAGEs) signalling pathway [71]. Additionally, DN has been associated with the induction of the NF-κB inflammatory pathway, ultimately resulting in podocyte injury and glomerulosclerosis in mice [57, 72]. Concurrently, D-ribose has been found to up regulate Bax expression, downregulate Bcl-2 expression, obstruct the Caspase 9/3 pathway, and promote mesangial cell apoptosis [55].

#### Diabetic encephalopathy

The association between diabetes and cognitive dysfunction dates back to 1922 [73]. Subsequently, the impact of DM on the central nervous system (CNS) has garnered significant attention. In comparison to non-diabetic controls, epidemiological evidence suggests that individuals with DM exhibit mild-to-moderate reductions in cognitive function, as assessed by neuropsychological testing [74]. In the initial 12 months following diagnosis, diabetic patients show a 10% prevalence of DN, a figure that escalates to as high as 50% after 25 years of diagnosis [75]. While T2DM predominantly affects the peripheral system and Alzheimer’s disease (AD) is characterized as a central nervous system ailment, both share commonalities owing to their chronic and intricate nature. They both involve oxidative stress and inflammation in their progression, which has led to the coining of the term "diabetes encephalopathy" or "type 3 diabetes" (T3DM) [76–78].

Uncontrolled diabetes increases the susceptibility to AD, and a possible link exists between glucose metabolism disorders and diabetes complicated by AD [79]. Several studies have demonstrated that D-ribose contributes not only to cognitive impairment and memory loss [80–83] but also plays a role in the onset of AD [58, 84]. Ribosylated BSA, incubated with ribose for approximately 7 days, exhibits the most pronounced cytotoxicity, resulting in a significant reduction in the viability of SH-SY5Y cells cultured for 24 hours [85]. The administration of D-ribose elevates formaldehyde concentration in the brains of mice and leads to cognitive dysfunction [86]. Moreover, D-ribose has been reported to activate Ca^2+^ /calmodulin-dependent protein kinase II (CaMKII), which catalyses the phosphorylation of Tau protein and consequently leads to cognitive impairment in mice [87].

RAGEs have been implicated in memory loss in rats, inhibiting brain-derived neurotrophic factor and tropomyosin-related kinase B (TrkB), ultimately resulting in Tau protein hyperphosphorylation [49, 88]. Additionally, levels of D-ribose, AGEs, Tau hyperphosphorylation, and neuronal cell death increase in the hippocampal CA4/DG region of streptozotocin-induced T1DM rats. Treatment with the transketolase (TKT) activator benfotiamine reduces TKT expression and activity in the brain, subsequently lowering D-ribose levels, albeit not D-glucose, and significantly reducing AGE accumulation [55]. Xu's study reveals that D-ribose can glycate neural cell alpha-synuclein (α-Syn), leading to the formation of molten globular aggregates that induce oxidative stress, resulting in heightened cytotoxicity [82].

## Conclusion

The debate regarding whether D-ribose is "beneficial" or "detrimental" has persisted for over half a century, yet a definitive answer remains elusive. This is due to the numerous inconsistencies and unknowns that persist in our understanding of D-ribose’s metabolism and biological functions. On one hand, D-ribose is considered a dietary supplement or pharmaceutical agent used to provide energy to the myocardium and skeletal muscles, with applications in the treatment of associated medical conditions. Conversely, in vitro studies have consistently reported cytotoxicity resulting from D-ribose’s potent nonenzymatic glycation capability.

Although the EFSA Panel on Dietetic Products, Nutrition and Allergies also established a general safe intake level for D-ribose, elevated D-ribose levels have been detected in both diabetic patients and animal models of diabetes, with strong associations with DN and diabetic encephalopathy [89]. Consequently, whether the supplementation of exogenous D-ribose proves advantageous or detrimental effects to cells and the human body necessitate comprehensive and systematic research on various aspects, including D-ribose’s impact on transmembrane transport, in vivo biodistribution, tissue-specific metabolism, metabolic regulation and dysfunction, its role in human diseases, and more. These efforts are vital for a full comprehension of D-ribose’s functionality, applications, and potential side effects.

Additionally, analytical techniques for quantifying D-ribose in vivo are of paramount importance in assessing its role in disease diagnosis. While some quantitative methods for detecting D-ribose in body fluids and cells have been developed [90–92], further refinements are needed to enhance sensitivity, accuracy, and practicality, enabling their clinical implementation.
